# Economic Impact of a Bluetongue Serotype 8 Epidemic in Germany

**DOI:** 10.3389/fvets.2020.00065

**Published:** 2020-02-14

**Authors:** Jörn Gethmann, Carolina Probst, Franz J. Conraths

**Affiliations:** Friedrich-Loeffler-Institut, Federal Research Institute for Animal Health, Institute of Epidemiology, Greifswald-Insel Riems, Germany

**Keywords:** Bluetongue disease, cattle, sheep, economy, gross margin analysis, disease control, costs

## Abstract

**Background and Objectives:** Germany was affected by Bluetongue virus serotype 8 (BTV-8) from 2006 to 2009 and recorded new cases since December 2018. We assessed the economic impact of the epidemic from the first cases in 2006 until 2018. Direct costs include production losses, animal deaths, and veterinary treatment. Indirect costs include surveillance, additional measures for animal export, disease control (preventive vaccination and treatment with insecticides), vector monitoring, and administration.

**Methodology:** To estimate the financial impact of BTV-8 on different species and production types at the animal level, we performed a gross margin analysis (GMA) for dairy and beef cattle, and sheep. To estimate the impact on the national level, we used a modified framework described by Rushton et al. (1) and applied a methodology described by Bennett (2). Both the GMA and the economic model on national level were implemented in Excel and the Excel Add-in @Risk. The tools, which are widely applicable, also for other diseases, are made available here.

**Results:** The financial impact of a BTV-8 infection at the animal level was estimated at 119–136 Euros in dairy cattle, at 27 Euros in beef cattle, and at 74 Euros in sheep. At the national level, the impact of the BTV-8 epidemic ranged between 157 and 203 million Euros (mean 180 million Euros). This figure consisted of 132 (73%) and 48 (27%) million Euros for indirect and direct costs. Indirect costs included 89 million Euros (67%) for vaccination, 18 million Euros (14%) for insecticide treatment, 15 million Euros (11%) for diagnostic testing of animals dispatched for trade, 8 million Euros (6%) for monitoring and surveillance, and 3 million Euros (2%) for administration. The highest costs were induced by a compulsory vaccination campaign in 2008 (51 million Euros; 28% of the total costs) and the disease impact on cattle in 2007 (30 million Euros; 17%).

**Discussion:** We compare the outcome of our study with economic analyses of Bluetongue disease in other countries, and discuss the suitability of GMA and the developed tools for a wider application in veterinary economics.

## Introduction

Bluetongue (BT) is a non-contagious infectious disease of domestic and wild ruminants caused by the BT virus (BTV), which belongs to the genus *Orbivirus* within the family *Reoviridae*. The virus is transmitted by biting midges of the genus *Culicoides*. At least 27 BTV serotypes have so far been detected worldwide (3). All ruminants are susceptible, although clinically apparent disease is most often reported in sheep. During the BTV-8 epidemic in 2006–2010 in Germany, clinical symptoms were observed in both, cattle and sheep. The most frequently reported signs were fever, weight loss, apathy, erosions of the oral mucosa, salivation, dysphagia, oedema of the head and lips, lameness, reduced milk yield and abortions (4–7). Effects on animal production in sheep and cattle for different BT serotypes and settings have been reviewed by Rushton and Lyons (8).

In August 2006, BTV-8 emerged for the first time almost simultaneously in Belgium, France, Germany, and the Netherlands (6, 9, 10). The disease hit an immunologically naïve and thus highly susceptible population. In Germany, the disease was first detected in late August 2006 (11). By the end of 2006, a total of 890 BTV-8 cases had been recorded in four German federal states and reported to the German Animal Disease Notification System (https://tsn.fli.de; public site TSIS: https://tsis.fli.de).

To determine the distribution and spread of BTV-8, the European Commission issued instructions for monitoring and surveillance in the member states of the European Union (SANCO/10581/2006 Rev 4). These included serological surveys and testing sentinel animals for antibodies to detect potential new cases as early as possible in 2007. Based on this working document, the European Commission regulation (EC) No 1266/2007 was launched, which established harmonized disease control measures, including preventive vaccination, a sentinel program, vector monitoring and monitoring in wild ruminants.

**Figure 1:**
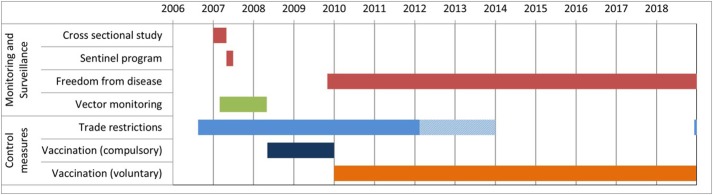
Timeline of official monitoring, surveillance, and control measures regarding BTV-8 in Germany.

The following measures were initiated in Germany (Figure 1): (i) a cross-sectional study (February to April 2007) within the 150 km restriction zone to assess the prevalence of BTV-8 infections in cattle and sheep (12); (ii) a sentinel program to detect the re-occurrence of BT, during which ~150 animals from 10 to 15 farms were monthly tested for antibodies to BTV-8 in each federal state; (iii) wildlife monitoring (2007 until today); (iv) vector monitoring to obtain information on the distribution and seasonal activity of potential BTV-8 vectors. Further disease control measures included the improvement of biosafety at the farm level, treatment of animals and stables with insecticides, and the testing of animals dispatched for trade.

Despite these measures BTV-8 re-occurred in May 2007. The disease spread over wide parts of Germany and affected more than 20,000 farms, causing the death of animals and substantial production losses, especially in sheep (11, 13–16).

As soon as commercial vaccines against BTV-8 had become available, they were tested for safety and efficacy (17, 18). Germany then initiated a country-wide mandatory vaccination campaign for cattle, sheep and goats, which started in May 2008. During 2008 and 2009, the number of outbreaks decreased sharply. Especially in the affected regions, farmers perceived vaccination positively. They realized that it prevented output losses and allowed trade and animal movements without restrictions. However, when vaccination became voluntary in 2010, farmers' willingness to vaccinate against BTV-8 was estimated at only 43% for cattle and 34% for sheep (19). Nevertheless, the BTV-8 epidemic subsided, so that Germany was declared officially free from BTV on 15 February 2012.

In August 2015, BTV-8 re-emerged in France (20) and in 2017 also in Switzerland (21). Compared to the strain that had circulated from 2006 to 2009, the current BTV-8 strain seemed to be less virulent, although the genome of the virus had remained stable (22, 23). In November 2017, a second BTV-serotype (BTV-4) was introduced to France (24). In view of the situation in the neighboring countries, the German federal state of Baden-Wuerttemberg encouraged farmers to vaccinate cattle, sheep and goats against both, BTV-8 and BTV-4, also by providing financial support. However, vaccination is not mandatory, and since the costs have to be borne mainly by farmers, the vaccine coverage was only about 25% by the end of 2018 (25). On 12 December 2018, two BTV-8-positive cattle were detected in Baden-Wuerttemberg as part of routine BTV surveillance (animals were tested by PCR and serological tests on December 6th; the test results were confirmed by the national reference laboratory; the outbreaks were recorded in the German animal disease notification system on 12th December 2018). The animals were clinically healthy. Again, monitoring was intensified and consigned animals could only be moved from this region to areas not under restriction, if the animals had been vaccinated against BTV-8 or tested for BT with a negative result (according to Article 8 of Commission Regulation (EC) No 1266/2007 serological or agent identification test).

There are ~11.8 million cattle in Germany (thereof about 40% dairy) and 1.6 million sheep (as of Mai 2019, Federal statistical office; https://www.destatis.de/). Livestock farming is the main source of income in agriculture in the country (26). Especially in the light of the re-emergence of BTV-8 and a potential future introduction of other BTV serotypes, namely BTV-4, disease contingency plans are evaluated, also from an economic point of view.

The purpose of this study was to carry out an ex-post economic impact analysis for BTV-8 in Germany for the years 2006–2018. The aim is to provide stakeholders and decision makers with a transparent evaluation of the potential benefits of preventing and controlling a vector-borne disease in livestock. The tools developed for the assessments and calculations are made available with this publication, so that they can be applied to other diseases and scenarios.

## Materials and Methods

**Figure 2:**
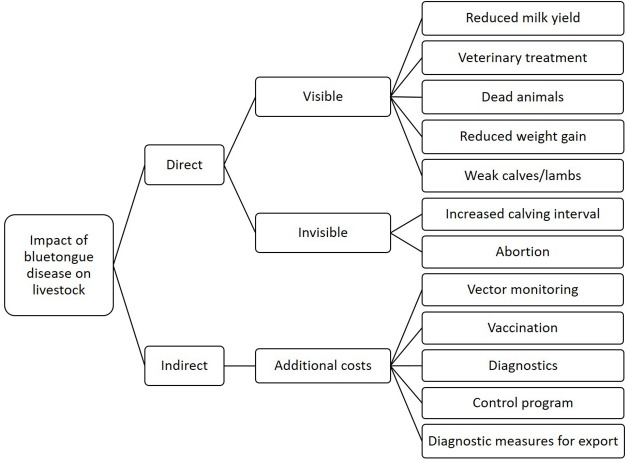
Impact of Bluetongue disease on cattle and sheep.

To calculate direct losses on the animal level, we used a gross margin analysis (GMA). To estimate the economic impact of BT on the national level, we applied a modified framework previously described by Rushton et al. (1, 27) (Figure 2) and a standardized method described by Bennett (2). The method can be adapted to BT as described elsewhere (28). Both, the GMA and the economic model run at the national level were implemented in a stochastic-deterministic spreadsheet in Excel version 2019 (Microsoft® GmbH, Unterschleißheim, Germany) and @Risk 7.0.0 (Palisade Corporation, Newfield, NY, USA). @RISK is an add-in to Microsoft Excel that allows analyzing risks using Monte Carlo simulation (https://www.palisade.com/). The spreadsheets for both, the GMA and the economic model, as well as user manuals, are provided in Supplementary Material.

The national average of herd performance as well as epidemiological and economic data were collected from Eurostat (https://ec.europa.eu/eurostat), the official German animal disease notification system (*TierSeuchenNachrichten-System*, https://tsn.fli.de/; public site: https://tsis.fli.de), the Federal statistical office (*Statistisches Bundesamt*, https://www.destatis.de/), the Identification and Information System for Animals (*Herkunftssicherungs- und Informationssystem für Tiere*, https://www.hi-tier.de/), and the Federal Office for Agriculture and Food (*Bundesanstalt für Landwirtschaft und Ernährung*, https://www.ble.de/). Other input parameters were obtained from the Bavarian State Agency for Agriculture (*Bayerische Landesanstalt für Landwirtschaft*, https://www.stmelf.bayern.de/idb/default.html), the German Association for Technology and Structures in Agriculture (*Kuratorium für Technik und Bauwesen in der Landwirtschaft e.V*., https://www.ktbl.de/), the animal health services of the federal states (*Tiergesundheitsdienste*), and the animal disease compensation funds of the federal states (*Tierseuchenkassen*). Values for the surveillance costs were obtained from reports of the federal states to the German Federal Ministry for Food and Agriculture and the annual applications of the Federal Ministry to the European Commission for co-financing animal disease control and surveillance (https://ec.europa.eu/food/funding/animal-health/national-veterinary-programmes_en). Parameters with fluctuation (e.g., milk yield, milk price) were resampled from known values with 10,000 iterations. Details are given in Supplementary Tables S1–S3. All monetary values are expressed in Euros.

### Economic Impact at the Animal Level

Direct costs at the animal level, i.e., production losses due to clinical illness (dC_py_), were estimated by calculating the difference between the gross margin (GM) of a healthy animal and the GM of a clinically ill animal. The GM was calculated per year (2006–2018) separately for dairy cattle (GM_Dy_), beef cattle (GM_Fy_), and sheep (GM_Sy_). Since infected calves and heifers were rarely reported to show clinical signs, we focused on adult animals. The GMA was performed according to standard procedures (https://www.stmelf.bayern.de/idb/default.html). To calculate the impact of BTV-8 at the animal level, we included the value of lost animal production with the disease-related intervention costs. Details of the GMA are provided in Supplementary Tables S1A (GM_Dy_), S1B (GM_Fy_), S1C (GM_Sy_). For example, GM_Dy_ was calculated as follows:

$$\begin{array}{l} GM_{Dy}=(R_{miy}+R_{any}+R_{may})-(VC_{ry}+VC_{fey}+VC_{cry}\\ \;+VC_{vy}+VC_{way}+VC_{insy}+VC_{macy}+VC_{laby}+VC_{misy}) \end{array}$$

i.e., including the revenues (R) for selling milk (R_miy_), animals (R_any_), and manure (R_may_), and the variable costs (VC) for restocking (VC_ry_), feed (VC_fey_), calf rearing (VC_cry_), veterinary treatment (VC_vy_), water/electricity (VC_way_), insemination (VC_insy_), machines (VC_macy_), hired labor (VC_laby_) and miscelaneous (VC_misy_).

For dairy cattle, parameters influenced by BTV-8 are associated with reduced fertility (prolonged calving interval due to abortion and stillbirth), lower milk yield, and costs for restocking (assuming the goal of maintaining the herd size constant despite increased mortality of calves and cows as the reference). In beef cattle, the main impact of BTV-8 was due to a drop in feed intake during the first days of disease, resulting in reduced daily weight gain and thus in a prolonged fattening period (assuming the goal of reaching the usual slaughter weight as the reference). For sheep, losses were mainly attributed to reduced revenues for selling animals (due to reduced slaughter weight) and the need to purchase new ewes (increased replacement rate resulting from death of ewes and lamb losses). In all animal species, the disease increased the costs for veterinary treatment. Details on the influence of BTV-8 on the GM are provided in Supplementary Tables S2A–C.

### Economic Impact on National Level

The results of the GMA were extrapolated from the animal level to the national population level separately for each species (cattle, sheep), production type (dairy, meat), and year (2006–2018). Details on the economic model are provided in Supplementary Table S3. The economic impact (net total costs) at the national level (C_BT_) includes direct (DC_y_) and indirect costs (IC_y_) and was calculated as follows:

$$\begin{array}{l} C_{BT}=\overset{2018}{\underset{y\;=\;2006}{\sum }}DC_{y}+\;IC_{y}\; \end{array}$$

#### Direct Costs

Direct costs at the national level per year (DC_y_) include production losses due to clinical illness (DCc_y_) and the value of the animals that succumbed to the disease (DCd_y_):

$$\begin{array}{l} DCy=DCc_{y}+DCd_{y}\; \end{array}$$

##### Production losses

To estimate the production losses caused by clinical illness (DCDc_y_, DCFc_y_, DCSc_y_), we multiplied the number of animals that had developed clinical signs with the average direct costs per animal (dCD_y_, dCF_y_, dCS_y_), which had previously been calculated in the GMA (which also included the veterinary costs). The number of animals that developed clinical signs were estimated by multiplying the number of newly infected cattle (nCi_y_) and sheep (nSi_y_) with the estimated morbidity for cattle (rcc) and sheep (rsc), and in case of cattle, with the proportion of dairy (pd) or beef (pf) cattle in the total cattle population.

$$\begin{array}{lll} DCDc_{y} & = & {nCi}_{y}\;*\;rcc\;*\;pd/100\;*\;dC_{py}\\ DCFc_{y} & = & {nCi}_{y}\;*\;rcc\;*\;pf/100\;*dC_{py}\\ DCSc_{y} & = & {nSi}_{y}\;*\;rsc\;*\;dC_{py} \end{array}$$

For cattle (rcc), morbidity was estimated to range between 5 and 15%, and for sheep (rsc), between 15 and 25%. The number of newly infected cattle and sheep per year were estimated as follows:

$$\begin{array}{l} nCi_{y}=\;\frac{I_{sy}}{100}\;*\;ncz_{y}\\ nSi_{y}=\;\frac{I_{sy}}{100}\;*\;nsz_{y} \end{array}$$

where I_sy_ is the yearly incidence per species (cattle, sheep), and ncz_y_ and nsz_y_ the number of cattle and sheep in the restriction zones.

To estimate I_sy_ for the year 2006, we used the results of a cross-sectional study performed in early 2007 (12). Since this study had revealed an underreporting of outbreaks (affected farms), we assumed that the relation between the numbers of officially reported outbreaks (P_y_) and I_sy_ remained constant over the years. I_sy_ was therefore estimated as follows:

$$\begin{array}{l} I_{sy}=\;\frac{I_{2006}}{P_{2006}}\;*\;P_{y} \end{array}$$

To estimate the numbers of newly infected cattle (nCi_y_) and sheep (nSi_y_), we multiplied the number of cattle (ncz_y_) or sheep (nsz_y_) in the restriction zones with the respective incidence. Regarding the number of animals in the restriction zones, we used the numbers of cattle and sheep kept in the BT-affected federal states for the year 2006, and the whole German cattle population for the years 2007–2011. Since 2012, no restriction zones for BT had remained.

##### Animal losses

To estimate the value of animals that succumbed to disease (DCd), the number of dead animals was multiplied with the compensation paid by the animal disease compensation fund for cattle (vc_y_) and sheep (vs_y_). The numbers of dead cattle (nCd_y_) or sheep (nSd_y_), respectively, were estimated based on data provided by the animal disease compensation funds and animal health services for the year 2007, assuming that the relation between newly infected (nCi_y_, nSi_y_) and dead animals (nCd_y_, nSd_y_) in 2007 (mean case-fatality ratio) remained constant throughout the years. Compensation includes the common value of the animals and for cattle also the disposal costs.

#### Indirect Costs

Indirect costs at the national level per year (IC_y_) include the costs for all legal provisions successively implemented to control BTV-8, including surveillance (ICS_y_), measures for animal export (ICE_y_), treatment with insecticides (ICI_y_), vaccination (ICV_y_), vector monitoring (ICM_y_) and administration time for establishing restriction zones and reporting (ICA_y_):

$$\begin{array}{l} IC_{y}=ICS_{y}+ICE_{y}+ICI_{y}+ICV_{y}+ICM_{y}+ICA_{y}\; \end{array}$$

##### Surveillance

The costs for BT surveillance according to SANCO/10581/2006 Rev 4 and Commission Regulation (EC) No 1266/2007 (ICS_y_) include the costs for the cross-sectional study performed in winter 2007, the sentinel program (early detection) performed in early 2007, and the BT monitoring for disease detection performed between 2007 and 2018. For cattle, sheep and goats, respectively, the costs for BT monitoring were calculated as follows:

$$\begin{array}{l} ICS_{y}=nfs_{y}\;*\;(ct_{f}+cp_{f})+ns_{sy}\;*\;csa_{s}+n_{ELI}\;*\;c_{ELI}\\ \;+n_{PCR}\;*\;c_{PCR} \end{array}$$

where nfs_y_ is the number of tested farms, ct_f_ and cp_f_ the travel and personnel costs per tested farm, ns_sy_ and csa_s_ the number of samples and sampling costs per species, n_ELI_ and n_PCR_ the number of samples tested by enzyme-linked immunosorbent assay (ELISA) or polymerase chain reaction (PCR), and c_ELI_ and c_PCR_ the respective costs for testing.

The numbers of tested farms and animals, as well as the costs for sampling and laboratory analysis were retrieved from the national applications for co-financing (Commission Decisions 2007/20/EC, 2008/655/EC, 2009/883/EC, 2010/712/EU, 2011/807/EU, 2012/282/EU, 2012/761/EU, 2013/722/EU, 2014/925/EU, 2014/288/EU, 2015/2444/EU, and 2016/969/EU). These documents are published at https://ec.europa.eu/food/funding/animal-health/national-veterinary-programmes_en.

Travel costs for official veterinarians (ct_f_) were estimated as follows:

$$\begin{array}{l} ct_{f}=k\;*\;d\;*\;2\; \end{array}$$

where k is the fee per km and d the average distance between the veterinary office and a farm.

Personnel costs for official veterinarians (cp_f_) were estimated as follows:

$$\begin{array}{l} cp_{f}=ts_{f}\;*\;cp_{h}\; \end{array}$$

where ts_f_ is the time spent on the farm and cp_h_ the average personnel costs of an official veterinarian per hour.

##### Measures for animal export

Following Commission Regulation (EC) No 1266/2007, Germany was between 2006 and 2008 requested to confirm that all animals intended for movement to BT-free EU member states or export to third countries were negative either in a BT-specific ELISA or by PCR. These additional costs for movement or export testing (ICE_y_) had to be borne by the farmers and were estimated as follows:

$$\begin{array}{l} ICE_{y}=(nce_{y}\;*\;pe_{y}\;*\;cet_{y})+(nse_{y}\;*\;pe_{y}\;*\;cet_{y}) \end{array}$$

where nce_y_ and nse_y_ are the numbers of cattle and sheep exported per year, pe_y_ the proportion of animals that were exported to BT-free countries and had therefore to be tested, and cet_y_ the test costs per animal. The costs caused by animal movements within the country were not taken into account, since almost all regions of Germany were part of a single restriction zone from 2007 until 2012.

##### Insecticide treatment

The costs for treatment with insecticides (ICI_y_) were calculated as follows:

$$\begin{array}{l} ICI_{y}=(ncz_{y}*ci_{y}+ncfz_{y}*ci_{fy})*pic_{y}+(nisf_{y}*\;s_{f}\\ \;*ci_{y})+(nisf_{y}*ci_{fy}) \end{array}$$

where ncz_y_ is the number of cattle in restriction zones, ci_y_ the costs for insecticides, ncfz_y_ the number of cattle farms in restriction zones, ci_fy_ the personnel costs per treated farm, pic_y_ the proportion of cattle (animals and farms) treated with insecticides, nisf_y_ the number of infected sheep farms and s_f_ the mean number of sheep per farm.

##### Vaccination

Depending on the animal species and the vaccine, two or three injections were required for a complete basic immunization. After that, the animals had to be re-vaccinated once a year. To calculate vaccination costs at the animal level for cattle (ICVc_y_) and sheep (ICVs_y_), we accounted for the costs per vaccinated animal and the costs per vaccinated farm. The costs per vaccinated animal were estimated by multiplying the number of vaccinations (cattle nvdc_y_ or sheep nvs_y_) with the sum of the costs for the vaccine (cattle cvc_d_ or sheep cvs_d_) and vaccination per immunization dose (cattle cvac_d_ or sheep cvas_d_). Since vaccines had to be applied by a veterinarian, vaccination costs at the farm level include the numbers of vaccinated farms (cattle nvcf_y_ or sheep nvsf_y_) and the herd fee charged by the veterinarian (cattle cc_f_ or sheep cs_f_) according to the following equations:

$$\begin{array}{l} ICVc_{y}=nvdc_{y}\;*\;\left(cvc_{d}+cvac_{d}\right)+\left(nvcf_{y}\;*\;cc_{f}\right)\\ \;ICVs_{y}=nvs_{y}\;*\;\left(cvs_{d}+cvas_{d}\right)+\left(nvsf_{y}\;*\;cs_{f}\right)\; \end{array}$$

##### Vector monitoring

The costs for vector monitoring (ICM_y_) account for the costs for vector traps and data loggers (cvmt_y_), trap management (cvmm_y_), and entomological tests (cvme):

$$\begin{array}{l} ICM_{y}=nvt_{y}*(cvmt_{y}+cvmm_{y})+nvme_{y}*cvme_{y} \end{array}$$

where nvt_y_ are the number of vector traps, and nvme_y_ the number of entomological tests.

##### Administration

The administration costs include the costs for epidemiological investigations (farm visits), for the establishment of restriction zones and the time required for reporting. Personnel and travel costs for farm visits of official veterinarians were also taken into account, so that calculations were performed according to the following equation:

$$\begin{array}{l} {ICA_{y}=tP}_{y}\;*\;d\;*\;k\;*\;2+\left(t_{f}\;*\;p_{h}\right)\; \end{array}$$

where tP_y_ ist the prevalence of BT (total number of affected cattle and sheep farms per year), d the average distance between the veterinary office and the affected farm, k the fee charged per driven km, t_f_ the average time spent per farm, and p_h_ the average personnel costs for an official veterinarian per working hour.

#### Sensitivity Analysis

We analyzed the effect of the input variables where we entered a distribution on the output mean (sensitivity analysis). The sensitivity analysis was performed in @Risk 7.0.0 (Palisade) with a one-at-a-time method (29, 30), where each variable is analyzed separately. The sensitivity analysis was carried out as follows (https://kb.palisade.com/index.php?pg=kb.page&id=248): (1) all iterations are ranked by ascending values of the input; (2) the ranked iterations are attributed to 10 bins (in this case with 10,000 iterations, each bin contains 1000 values); (3) the mean of the output values of each bin is computed; (4) 10 output means from the bins are compared. The lowest output mean gets the number at the left edge; the highest of the 10 output means receives the number at the right edge.

Finally, the results of the sensitivity analysis were assessed qualitatively using a tornado plot, which shows how the mean of the model varies over the range of each input variable.

## Results

### Economic Impact on Animal Level

For the dairy sector, direct costs ranged between 119 and 136 Euros per infected animal, depending on the milk price (Table 1). Most of the costs resulted from the need to restock (99 Euros/animal), veterinary treatment (26 Euros) and production losses (24 and 18 Euros less output for milk and calf sales, respectively). In the beef sector, direct costs amounted to 27 Euros per animal on average. They were mainly attributable to the prolonged fattening period. For sheep, direct costs were estimated at 74 Euros per animal on average. They were mainly due to reduced revenues for lamb sales (59 Euro per infected ewe) and veterinary treatment, especially after abortions (10 Euros/animal) (data not shown).

**Table 1:** Direct costs of a BTV-8 infection per animal, with minimum, mean, maximum, 5 and 95% percentiles in million Euros.

**Gross margin**	**Minimum**	**Mean**	**Maximum**	**5%**	**95%**
Dairy 2006	78	122	515	92	173
Dairy 2007	78	129	928	94	194
Dairy 2008	90	136	614	102	198
Dairy 2009	79	119	391	91	164
Beef (2006–2009)	14	27	40	22	33
Sheep (2006–2009)	42	74	104	60	88

### Economic Impact on National Level

The net total costs of the BTV-8 epidemic in Germany, including prevention and control measures over the last 13 years (2006–2018), ranged between 157 and 203 million Euros (mean 180.4 million Euros, standard deviation 6 million) (Table 2). This figure includes on average 132.1 (73%) million Euros indirect and 48.3 (27%) million Euros direct costs.

**Table 2:** Minimum, maximum and mean net costs (in million Euros) of BTV-8 in Germany from 2006 to 2018) with 5% and 95% percentiles.

**Cost factor**	**Minimum**	**Mean**	**Maximum**	**5%**	**95%**
Net total costs	157.002	180.406	202.995	169.915	191.056
Total direct costs	37.091	48.313	60.842	42.403	54.554
Direct costs cattle	27.756	37.449	50.226	31.797	43.372
Direct costs sheep	7.482	10.864	14.893	8.802	12.965
Total indirect costs	115.836	132.092	149.548	123.500	140.840
Vaccination cattle	64.148	74.497	85.153	67.521	81.543
Vaccination sheep	12.996	14.064	15.187	13.450	14.682
Insecticide treatment cattle	12.518	16.894	21.176	14.476	19.269
Insecticide treatment sheep	805	1.078	1.349	926	1.233
Export measures cattle	7.153	12.263	20.769	8.773	17.047
Export measures sheep	1.782	2.627	3.431	2.183	3.069
Monitoring and surveillance	7.562	7.882	8.225	7.700	8.070
Administration	1.755	2.788	3.917	2.171	3.421

*Totals and subtotals are indicated by gray shading*.

Mean indirect costs included 106.5 million Euros for disease control measures (vaccination and insecticide treatment, 59% of the net total costs), 14.9 million Euros for additional measures relating to export (12.3 million only for cattle), 7.9 million Euros for BT monitoring and surveillance (including 1.2 million Euros for vector monitoring in 2007 and 2008), and 2.8 million Euros for administration. Disease control measures consisted of 88.6 million Euros for vaccination (74 and 14 million Euros for cattle and sheep, respectively) and 18.0 million Euros for treatment with insecticides (16.9 and 1.1 million Euros for cattle and sheep) (see Tables 2, 3).

**Table 3:** Mean net total costs of BTV-8 in Germany per year from 2006 to 2018 (in million Euros).

	**2006**	**2007**	**2008**	**2009**	**2010**	**2011**	**2012**	**2013**	**2014**	**2015**	**2016**	**2017**	**2018**
Net total costs	9.250	59.105	66.810	27.022	10.441	5.910	1.453	0.177	0.076	0.162	2.358	2.192	1.528
Total direct costs	1.863	39.765	6.664	0.022	0	0	0	0	0	0	0	0	0
Direct costs cattle	1.461	29.661	6.308	0.020	0	0	0	0	0	0	0	0	0
Direct costs sheep	0.402	10.105	0.356	0.002	0	0	0	0	0	0	0	0	0
Total indirect costs	7.387	19.339	60.146	27.001	10.441	5.910	1.453	0.177	0.076	0.162	2.358	2.192	1.528
Insecticide treatment cattle	2.893	7.959	2.063	1.021	1.002	0.982	0.974	0	0	0	0	0	0
Insecticide treatment sheep	0.040	1.002	0.035	0	0	0	0	0	0	0	0	0	0
Vaccination cattle	0	0	44.530	17.284	7.916	4.429	0.305	0.031	0.001	0.001	1.462	1.691	1.199
Vaccination sheep	0	0	6.777	5.549	1.358	0.344	0.032	0.003	0	0	0.740	0.386	0.209
Export measures	3.500	5.047	3.929	2.415	0	0	0	0	0	0	0	0	0
Administration	0.190	2.273	0.324	0.001	0	0	0	0	0	0	0	0	0
Monitoring and surveillance	0.765	3.058	2.488	0.730	0.165	0.155	0.142	0.143	0.075	0.161	0.156	0.115	0.119

*Totals and subtotals are indicated by gray shading*.

Mean direct costs mainly arose in the cattle sector (37.4 million Euros, 21% of the net total costs) (Table 2). In the sheep sector, they amounted to 10.9 million Euros (6%). Direct costs were highest in 2007, when they reached 39.8 million Euros (29.7 million in cattle, 10.1 million in sheep) (Table 3). In 2007, the animal compensation funds paid for 10,240 cattle and 33,233 sheep that prematurely died due to BTV-8 infection. This corresponds to a mortality ratio of 0.081 for cattle and 1.4 for sheep. The compensation paid per animal was 1,500–1,900 Euros for cattle and 120–170 Euros for sheep, including rendering costs. This corresponds to total compensation payments of 17.3 and 4.2 million Euros for cattle and sheep in Germany.

**Figure 3:**
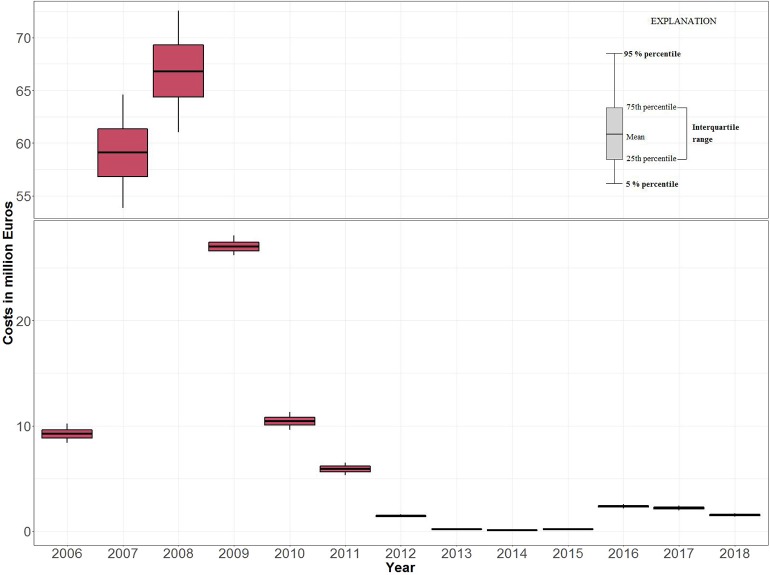
Boxplots of the net total costs of BTV-8 in Germany (in million Euros) with mean values, 25%–75% (box) and 5%–95% percentiles.

**Figure 4:**
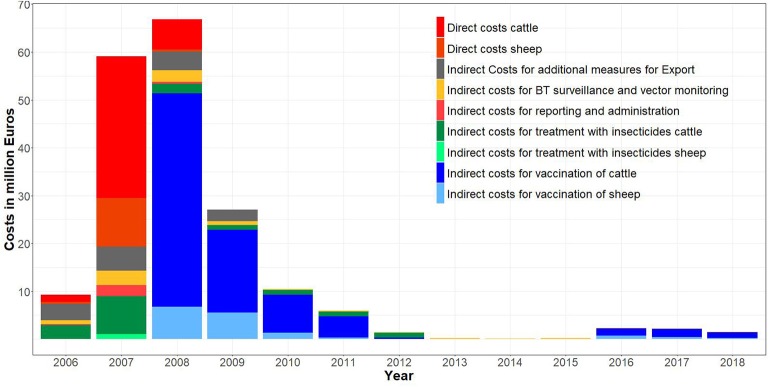
Net total costs of BTV-8 in Germany per year from 2006 to 2018 per cost factor (mean costs by cost factors).

The yearly costs were highest in 2008 and 2007, with 66.8 (37% of net total costs) and 59.1 million (32%) Euros, respectively (Table 3 and Figure 3). After peaking in 2008, they gradually dropped from 27.0 million (2009) to 74 thousand Euros (2014). In 2015, they started to increase again and reached 1.5 million Euros in 2018 (Table 3 and Figure 4).

In 2007, the total costs were mainly attributable to direct costs caused by BTV-8 infections. In 2008, total costs accrued mainly from vaccination (51.3 million Euros, thereof 44.5 million Euros for cattle, 25% of the total costs) (Table 3). In 2009 and 2010, vaccination of cattle cost 17.3 and 7.9 million Euros (10% and 5%), respectively. Since 2010, the costs for voluntary vaccination had to be borne by the farmers, so that the number of vaccinations decreased and in 2014–2015, almost the entire costs consisted of the expenditures for monitoring and surveillance. Since 2015, financial incentives were used in the south-west of Germany (mainly Baden-Wuerttemberg) to motivate farmers to participate in voluntary vaccination, so that the vaccination costs started to increase. Since 2016, again almost all investments went into vaccinating cattle (Figure 4).

Between 2012 and 2018, no animal trade restrictions were in place, but monitoring and surveillance were still carried out. These measures caused costs of about 150 thousand Euros in 2013–2015. In 2015, monitoring and surveillance costs increased due to the voluntary vaccination program (Table 3 and Figure 4).

**Figure 5:**
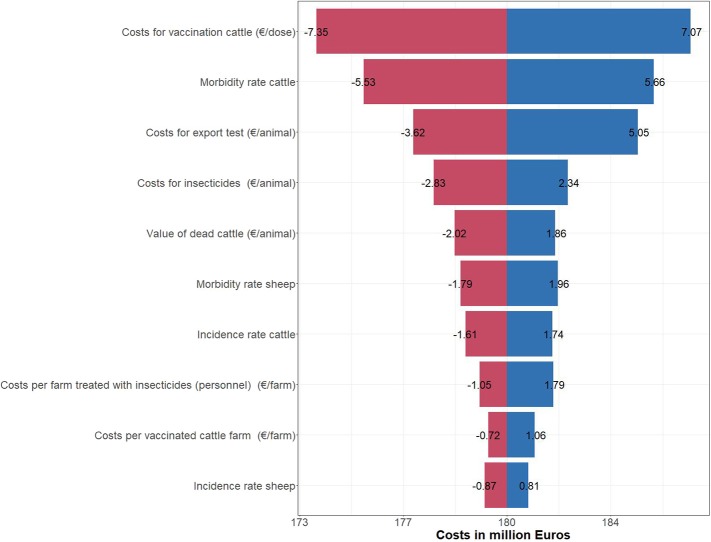
Tornado plot for the ten most relevant cost factors of BTV-8 in Germany from 2006 to 2018. The red and the blue bars show the change in the means, when changing the single parameter.

Sensitivity analysis showed that the proportion of infected animals that develop clinical signs and the impact on milk yield were strongly related to the veterinary treatment costs and production losses. The costs per vaccination dose had the strongest impact on the indirect costs (Figure 5).

## Discussion

When BTV-8 emerged in Germany in 2006, no validated contingency plans were available, because the disease had never before occurred in the country and came completely unexpected. Within a short time, BTV-8 had a substantial impact on animal health in the affected livestock species and far-reaching consequences on animal trade, causing severe economic losses. According to our calculations, direct costs amounted to 40 million Euros within a single year (2007). While the direct costs were mainly borne by the farmers, the main proportion of the costs (vaccination) had to be covered by the animal disease compensation funds of the German federal states, and were co-financed by the European Union in 2008 and 2009.

To capture the full impact of BTV-8, we chose a study period of 13 years, starting in 2006, when BTV-8 first emerged in Germany, and ending in 2018, 6 years after the disease-free status was re-gained. Yet, since 2018, a new voluntary vaccination campaign is in place in western and southern regions of Germany, due the evolving BT-situation in neighboring countries, in particular Belgium, France and Switzerland.

Our study supports the view that the measures taken in reaction to a disease sometimes have a greater financial impact than the production losses caused by the disease itself (8). With 74 million Euros, the compulsory vaccination campaign in 2008–2009 was the largest cost factor throughout the study period. On the other hand, it was successful in eradicating a disease that caused not only economic damage, but also substantial suffering in infected animals.

After the initial introduction of BTV-8 in 2006, the last outbreak occurred in Germany in November 2009 and the country re-gained the official BT-free status 26 months later, in February 2012. Possibly, the numbers of new infections and thus the direct costs might have decreased anyway due to immunity after natural infection, even without vaccination. Yet, we still regard compulsory vaccination as economically beneficial, as it substantially contributed to reducing virus circulation and eventually to eradicating the disease within a short period (31). Therefore, in certain epidemiological situations, financial support should be provided to farmers who are willing to vaccinate their animals as a measure to prevent clinical BT and to reduce the size of the susceptible population, which may help to limit the spread of the disease at least to some extent.

To assess the costs of BTV-8 at the animal level, we used the GMA because it is widely used by farmers and can be easily adapted to assess the economic impact both, at the animal and farm level, for any disease with clinical symptoms.

Uncertainty regarding some input parameters limited the accuracy of our estimates. A number of key input parameters for the GMA had to be derived from expert opinion. Due to the large variation in BTV-8-associated morbidity, mortality and the severity of clinical disease, as well as large price differences for milk and vaccination (performed by both private and official veterinarians) between different regions, farms and years, our estimations were highly variable. Therefore, our model might have under- or overestimated the total costs. A sensitivity analysis identified the parameters that had the highest impact on the model outcome (e.g., morbidity, disease effects on cattle and sheep; Figure 4).

Not only animal and farm-related variables, but also official data were sometimes difficult to parametrize. For example, the annual reports to the European Commission underwent several changes, e.g., regarding measures eligible for co-financing or maximum financial contribution limits for testing, and they do not display the data every year in the same format. For example in 2009, costs for sheep and goats could not be differentiated, so missing data had to be estimated by interpolating the values from the previous and following years.

Moreover, we could not address all factors that might have had an economic effect on BT and its control. For example, we could not include the costs or returns from investing in biosecurity due to lack of information. We could also not include the time, farmers spent on handling diseased animals, which could have been invested in other productive activities, because this time is not known and could not be reliably estimated. Moreover, BT caused significant animal welfare problems, which were not included in our study, as animal welfare does not generate profit in terms of money and animal welfare problems are difficult to translate into monetary losses. Although Germany is highly dependent on international trade in animals and animal products, losses due to trade restrictions were only included as far as the additional testing for BT is concerned that had to be performed according to EU legislation. Further effects, for example trade partners' possible reactions to the country losing its BT-free status were not included, because the respective costs were not known and could not be reliably estimated. Moreover, we did not include additional expenditures caused by trade restrictions within Germany between August 2006 and September 2007, since the 20 and 150 km restriction zones changed frequently in short intervals (days or weeks). The fact that these parameters were not included in the calculation may have led to an underestimation of costs in 2006 and 2007. Despite these limitations, our results indicate that Germany has benefited from re-gaining its BT-free status within a short time and that the country is likely to benefit again from vaccination in the event of a new BT epidemic.

The economic impact of the BTV-8 epidemic has so far been assessed for Switzerland (32) and the Netherlands (NL) (16, 33). The cost-effectiveness of various possible surveillance systems for BT in Switzerland has also been evaluated (34) and an economic evaluation of the vector monitoring programmes in Austria and Switzerland has been conducted (35). These studies had different underlying questions and aims and therefore differed in the applied methods. All approaches have their specific advantages and disadvantages, but, overall, the variability in the methods used in the studies make it difficult to compare the outcomes. In addition, the epidemiological situation in Austria, Switzerland and Germany was not comparable, especially regarding farm structure, number of affected farms and animals, and control strategies, although the legislation in the field of animal health is similar in these countries as they are EU member states (Austria and Germany) or apply EU legislation (Switzerland).

Livestock production and the epidemiological situation regarding BT in Germany is most likely comparable to the conditions in the Netherlands. The Netherlands and Germany are part of the Common Market of the European Union. Landscape structures on both sides of the border between the two countries are similar. The epidemiological situation regarding Bluetongue disease was comparable, as the BTV-8 epidemic started in the border area between Belgium, Germany and the Netherlands in 2006 and spread rapidly in the entire region, reaching a high prevalence in the affected area of these countries (13). Farmers tend to keep more cattle per farm in the Netherland [159.9 cattle compared to 101.7 cattle in Germany (36)], while the predominant cattle breeds are similar in both countries. The main affected regions in Germany after 2006 were in the Northeast (North Rhine-Westphalia, Lower Saxony). Farm sizes and structures in this area of Germany are more similar to those in Belgium and the Netherlands as compared to the south of Germany, in particular Bavaria and Baden-Wuerttemberg.

Nonetheless, our cost estimations are relatively low as compared to the Dutch figures, especially when considering that the number of cattle in Germany is about three times higher than in the Netherlands. For 2006, we estimated a financial impact of about 9 million Euros, compared to 28–32 million Euros in the Netherlands (16, 33). In 2007, we estimated costs of about 59 million Euros, compared to 49 million Euros (33) and 164–175 million Euros (16), which is at least in part due to the differences in the applied methods. For Switzerland, the total BTV-8 disease costs including cantonal response measures have been estimated at 12.2 and 3.6 million Euros for 2008 and 2009 (32). Again, our estimations seem to be comparatively low (6.9 million and 21.000 Euros in 2008 and 2009), especially when considering that the number of cattle in Germany is about eight times higher than in Switzerland. Based on the data of the German animal compensation funds, we estimated the mortality ratio at the population level at 0.081. This is slightly lower than the estimate for mortality used by Häsler et al. (32). A study by the Scottish Government modeled the economic impact of different incursion scenarios and estimated the direct costs of BT, including reduced milk production, weight loss, mortality, veterinary treatment and testing at £ 30 million per year (37).

The main difference between our analysis and previous studies conducted for other countries consists in the estimation of the direct costs (production losses and veterinary treatment), which was relatively low (119–136 Euros per animal) in our assessment. In contrast to the study of Velthuis et al. (16), we estimated higher milk losses, but lower veterinary treatment costs and a lower morbidity. In the Dutch study, BTV-8 was assumed to decrease milk production by 5.4 kg/day for a period of 10.5 days, which resulted in a total decrease in milk production of 56 kg per infected cow (16). Another study from the Netherlands assumed the milk production in a BTV-8 infected cow to decrease by 51 to 52 kg, which corresponds to 0.3 to 0.9% of the annual production (38). In a further study, the losses for the reduction in milk production in the Netherlands were estimated to range between 3 and 94 (average 48) Euros per cow (39). A study that analyzed data of the BTV-8 epidemic in France for 2007 found that cows lost a mean of 1.2–3.4% (111–249 kg) of their total annual milk yield (40). This was higher than the losses estimated in our study, where we assumed a reduction in milk yield of about 100 kg per infected cow.

Regarding morbidity, Velthuis et al. (16) estimated 5% on the total population level and 88% for infected animals. By contrast, we estimated the number of infected (i.e., antibody-positive) cattle at about 1.29% on the national level (6.6% in the affected region) based on the results of a cross-sectional study conducted in 2007 (12). Assuming that only 5–15% of the infected animals show clinical signs, the morbidity on the population level was estimated at 0.66% (0.33–0.99%). If we had taken the Dutch morbidity figures, the direct costs for cattle in Germany would have reached 11–71 million Euros in 2006 or 13–308 million Euros in 2007. In our model, we originally used a morbidity value that is lower than the one in the model for the Netherlands. For comparison, we also recalculated our model using the morbidity rates mentioned in the publication on the Dutch data. In contrast to the Dutch studies, we did not include the costs for indoor-housing, which had the highest impact for sheep and goats in the Netherlands in 2006 (18 million Euros) (16, 33). Indoor housing was hardly practiced in Germany, although it was recommended or even required by veterinary authorities for some time early after the introduction of BTV-8 in 2006 while it was clear that indoor housing is not an effective control measure as the main Palearctic *Culicoides* vectors for BTV-8 (*C. obsoletus* and *pulicaris* complex) were found to occur also indoors (41, 42).

Regarding mortality, cow mortality ratios of 1.2, 1.3, and 1.4 for the age categories <3 days, 3 days−1 year, and >1 year, respectively have been reported in the BTV-8 epidemic in the Netherlands for 2007 (43). In our study, we assumed mortality ratios of 0.02 in adult cows and 0.03 in calves.

Regarding fertility, infected cows were five times more likely to return to service (RTS) within 56 days after the first insemination compared to non-infected cows in Dutch herds (44). The difference in time between the first and the last insemination was 101.6 days. Comparing exposed and non-exposed farms in France, RTS increased by 8–21% (45). In another study, the same authors reported an average effect of BTV-8 exposure with a 6.7% increase in RTS and 1.9% increase in short gestations (46). Regarding sheep (47), investigated an outbreak in a flock of 355 ewes in Belgium and detected an increased ratio of 15.7% in abortions. In addition, the authors found a reduction in fertility from 59–75% to 30%. Since the calving interval can directly be used in the GMA, we used this parameter (which was assumed to be prolonged by about 80 days), instead of the RTS.

For Austria, the total net costs of the BTV-8 surveillance and vaccination programmes 2005–2013 were estimated at 22.8 million Euros (48). In the same period, surveillance and vaccination cost 96.6 million Euros in Germany. This sum is relatively low, considering that the number of cattle in Germany is about six times higher than in Austria. Regarding vector monitoring, the costs for the period 2006–2010 have been estimated at 1.42 million Euros for Austria and 94,000 Euros for Switzerland (35). In Germany, vector monitoring was only performed in 2007–2008, incurring total costs of 1.2 million Euros.

In conclusion, our study shows that the BTV-8 epidemic caused high direct costs in Germany in 2007 and high indirect costs for the compulsory vaccination programme in 2008–2009. The measures taken in reaction to the emergence of BT had a greater financial impact than the production losses caused by the disease itself. It should be pointed out, however, that vaccination proved effective with regard to disease eradication within a short time, thus reducing the suffering of animals and allowing international trade without restrictions.

The tools we developed are widely applicable for analyzing the economic impact of livestock diseases at both, the animal and the national level. They were implemented in widespread software (Excel and @Risk), so that they can be easily used, also by decision makers without programming skills. The use of GMA for assessing the economic impact at the animal or farm level ensures that we speak “the same language” as farmers who are used to communicate with GMA figures, when they analyse and discuss their economic situation. @Risk (Palisade) is an Excel add-in that allows using distributions rather than fixed values, i.e., stochastic modeling. Furthermore, @Risk supports the analysis of stochastic models. It is easy to use for persons who are familiar with Excel and is also used by other groups for economic analysis [e.g., (49)], which may eventually make it easier to compare results. A disadvantage is that @Risk is not freely available and for some statistical methods, no reference is given (e.g., sensitivity analysis).

We developed an economic model to calculate the direct and indirect costs BTV-8 for the years 2006–2018 in Germany, a country where BTV-8 has been successfully eradicated in the past. The model may assist stakeholders and decision makers in the planning of future control strategies. The results of the model may be useful to decide on further preventive and control measures in the current BTV-8 epidemic in Germany. The model may also be adapted for other countries and other vector-borne diseases.

## Data Availability Statement

All datasets generated for this study are included in the article/Supplementary Material.

## Author Contributions

JG, FC, and CP contributed conception and design of the study. JG and CP developed and performed the economic analyses. CP, FC, and JG wrote the manuscript.

### Conflict of Interest

The authors declare that the research was conducted in the absence of any commercial or financial relationships that could be construed as a potential conflict of interest.
