# Supplementary material for: Economic Impact of a Bluetongue Serotype 8 Epidemic in Germany
Source: Front Vet Sci. 2020 Feb 14;7:65. doi: 10.3389/fvets.2020.00065 (PMC7034324; doi:10.3389/fvets.2020.00065)
Supplement: Supplementary file 5 [file Table_5.docx]

# **User manual for the Excel spreadsheet “Economic model”**

This manual aims at explaining the structure and use of the risk model spreadsheet developed for the simulation of the economic impact BTV-8 on population level. The spreadsheet was designed in Excel, using functions of the @Risk software (Palisade ©). Users need to have @Risk installed. For users who do not wish to use @Risk, we designed an Excel file named “Economic model_BT_without_aR”. In this case, the model uses fixed values instead of distributions, i.e. it is deterministic and not stochastic as the one implemented using @Risk.

The excel file includes default values for an **example,**  i.e. the BTV-8 epidemic in Germany. The values do not necessarily reflect the true situation. If you use @Risk, you can adapt all values in the white and blue cells according to your own observations and with your own data.

The Excel file contains nine interconnected spreadsheets:

1. The spreadsheet “**overview**” is the model outcome. It provides an overview of all cost factors of BTV-8 on population level. Further information about data sources, equations, distributions and references to documents containing the data used to define the input parameters can be found in the Supplementary Word document of this publication.
2. The spreadsheet “**indirect costs**” summarizes the indirect costs. It includes distributions (blue cells), calculations (yellow cells), and references to other cells (green cells).
3. The spreadsheet “**general Data cattle**” gives an overview on the general data of cattle. It includes distributions (blue cells), calculations (yellow cells), and references to other cells (green cells).
4. The spreadsheet “**cattle direct costs**” summarizes all factors used to calculate the direct costs for cattle. It includes distributions (blue cells), calculations (yellow cells), and references to other cells (green cells).
5. The spreadsheet “**general indirect costs**” summarizes all factors used to calculate the indirect costs in cattle. It includes distributions (blue cells), calculations (yellow cells), and references to other cells (green cells).
6. The spreadsheet “**sheep general data**” gives an overview on the general data of sheep. It includes distributions (blue cells), calculations (yellow cells), and references to other cells (green cells).
7. The spreadsheet “**sheep direct costs**” summarizes all factors used to calculate the direct costs for sheep. It includes distributions (blue cells), calculations (yellow cells), and references to other cells (green cells).
8. The spreadsheet “**sheep indirect costs**” summarizes all factors used to calculate the indirect costs for sheep. It includes distributions (blue cells), calculations (yellow cells), and references to other cells (green cells).
9. The spreadsheet “**Diagramm2**” displays a diagram of the mean costs per year including the sectors of the costs.
