# Supplementary material for: Economic Impact of a Bluetongue Serotype 8 Epidemic in Germany
Source: Front Vet Sci. 2020 Feb 14;7:65. doi: 10.3389/fvets.2020.00065 (PMC7034324; doi:10.3389/fvets.2020.00065)
Supplement: Supplementary file 6 [file Table_6.docx]

# **User manual for the Excel file “Gross margin analysis”**

This manual aims at explaining the structure and use of the risk model file developed for the simulation of the gross margin analysis for BTV-8 on **animal level**. The spreadsheets were designed in Excel, using functions of the @Risk software (Palisade ©). Users need to have @Risk installed. For users who do not wish to use @Risk, we designed an Excel file named “Gross margin_dairy_BT_withoutaR”. In this case, the model uses fixed values instead of distributions, i.e. it is deterministic and not stochastic as the one implemented using @Risk.

The excel file includes default values for an **example, i.e.** dairy cows of the breed Holstein Friesian in an average dairy farm in Germany. If you use @Risk, you can adapt all values in the white cells according to your own observations and with your own data. Blue cells include distributions, and grey cells calculations.

The Excel file contains four interconnected spreadsheets:

1. The spreadsheet “**gross margin**” is the main model. It summarizes all model inputs and parameters used to calculate the gross margin for both, a healthy and a diseased dairy cow. It also includes some comments on the input data. Further information on data sources, equations, distributions and references to documents containing the data used to define the input parameters can be found in the Supplementary Word document of this publication. In column G (red cells), you can adapt disease effects. The gross margin (in € per cow per year) for a **healthy** animal is displayed in cell **E46** and for a **diseased** animal in cell **I46**. The difference between both animals can be found in cell **E48**.
2. The spreadsheet “**feed costs**” contains detailed data used to calculate the feed costs for a healthy dairy cow. In our example, we assumed that an animal receives the amount of concentrate feed that is necessary to produce the expected amount of milk (milk yield). Based on the milk yield given in cell E10, the required amount of concentrate feed is calculated.
3. In the spreadsheet “**feed costs diseased**”, the feed costs for a diseased animal is calculated in a similar way as for a healthy animal.
4. The spreadsheet “**milk prices**” was added to take the high variability in the milk price in Germany into account. If you do not have the information on the milk price, you can add a distribution or a specific value in cell E13 in the spreadsheet “gross margin”.
