# Supplementary material for: Economic Impact of a Bluetongue Serotype 8 Epidemic in Germany
Source: Front Vet Sci. 2020 Feb 14;7:65. doi: 10.3389/fvets.2020.00065 (PMC7034324; doi:10.3389/fvets.2020.00065)
Supplement: Supplementary file 7 [file Data_Sheet_1.pdf]

Supplementary Material

1 Supplementary Tables

Table S1: Variables used in the Gross Margin Analysis (GMA) for healthy dairy cattle (A), beef cattle (B), and sheep (C) per animal. Normal distributions denote a mean value µ and the standard deviation σ in brackets; Triangle distributions denote a distribution with lower and upper limits and mode. Bold letters in the variables are used in the notations. To avoid duplicate notations, we combined upper and lower case characters.

(A) Dairy cattle

Variable	Unit	Notation	Calculation / value	Data source/ comments
**G**ross **M**argin healthy **D**airy per **y**ear	€	GM_{Dy}	$R_{Dy} - VC_{Dy}$	
**R**evenues **D**airy per **y**ear	€	R_{Dy}	$R_{miy} + R_{any} + R_{may}$	
**R**evenues for selling **mi**lk	€	R_{miy}	$my_y * ms_y * mp_m$	
**m**ilk **y**ield	kg/cow	my_y	Normal distribution (µ=yearly national average milk yield; σ=yearly mean standard deviation)	Monthly milk yield per federal state. Source: Federal statistical office, *Statistisches Bundesamt*; https://www.destatis.de/ Retrieved in June 2013
proportion of **m**ilk **s**old	per cent	ms_y	0.95	Fixed value. Source: Bavarian State Agency for Agriculture, *Bayerische Landesanstalt für Landwirtschaft*; https://www.stmelf.bayern.de/idb/milchkuhhaltung.html Retrieved in June 2013
**m**ilk **p**rice	€/kg	mp_{2006}	Sampling from milk prices in Germany 2006 (RiskResample)	Sampling of monthly average milk prices per federal state. Source: Federal Office for Agriculture and Food, *Bundesanstalt für Landwirtschaft und Ernährung*; https://www.ble.de/DE/BZL/Daten-Berichte/Milch-Milcherzeugnisse/milch-milcherzeugnisse_node.html Retrieved in June 2013
		mp_{2007}	Sampling from milk prices in Germany 2007 (RiskResample)	
		mp_{2008}	Sampling from milk prices in Germany 2008 (RiskResample)	
		mp_{2009}	Sampling from milk prices in Germany 2009 (RiskResample)	
**R**evenues for selling **an**imals	€	R_{any}	$R_{calfy} + R_{cowy}$	
**R**evenues from **calf** sale	€	R_{calfy}	$CC_y * p_{mcy} * r + CC_y * p_{fcy} * (1 - r)$	
number of **C**alves per **C**ow per year	n	CC_y	$\frac{365}{Ci} - \frac{365}{Ci * cm_y}$	
**p**rice **m**ale **c**alf	€	p_{mcy}	Triangle distribution (82; 124; 107)	Kloepfer et al. (2012)

**p**rice **f**emale **c**alf	€	p_{fcy}	Triangle distribution (31; 62; 45)	
male/female **r**atio	per cent	r	0.5	Fixed value, Bavarian State Agency for Agriculture
**R**evenues from **cow** sale	€	R_{cowy}	$(pC * wC - mC) * (1 - Cm) * RR$	
**p**rice slaughter **C**ow	€/kg	pC	2.05	Fixed value, Bavarian State Agency for Agriculture
average **w**eight of a slaughter **C**ow	kg	wC	326	Fixed value, Bavarian State Agency for Agriculture
**m**arketing costs of a **C**ow	€	mC	21	Fixed value, Bavarian State Agency for Agriculture
adult **C**ow **m**ortality rate	per cent	Cm_y	0.054	Fixed value, Bavarian State Agency for Agriculture
**R**eplacement **R**ate	per cent	RR	0.33	Fixed value, Bavarian State Agency for Agriculture
**C**alving **i**nterval	days	Ci	Triangle distribution (376; 417; 458)	Estimated. Source: Identification and Information System for Animals, *Herkunftssicherungs- und Informationssystem für Tiere* , HIT; https://www.hi-tier.de/
**c**alf **m**ortality rate	per cent	cm_y	Triangle distribution (5; 10; 15)	Bavarian State Agency for Agriculture
**R**evenues for selling **ma**nure	€	R_{may}	111	Fixed value, Bavarian State Agency for Agriculture
**V**ariable **C**osts healthy **D**airy per **y**ear	€	VC_{Dy}	$VC_{ry} + VC_{fey} + VC_{cry} + VC_{vy} + VC_{way} + VC_{insy} + VC_{macy} + VC_{laby} + VC_{misy}$	
**V**ariable **C**osts for **r**estocking	€	VC_{ry}	$(p_h + m_h) * RR$	
**p**rice for a **h**eifer	€	p_{hy}	1,950	Fixed value, Bavarian State Agency for Agriculture
**m**arketing costs for a **h**eifer	€	m_{hy}	33	Fixed value, Bavarian State Agency for Agriculture
**V**ariable **C**osts for **fe**ed	€	VC_{fey}	$\frac{my_y - my_{fy}}{ef_{con}} * p_{con}$	Feed costs included the costs for concentrates. For dairy cattle, estimations were based on the average daily intake depending on the amount of milk produced
**m**ilk **y**ield from **f**orage	kg	my_{fy}	$(E_{fy} - E_{by})/3.3$	3.3= Energy requirement per kg milk in MJ/kg; Bavarian State Agency for Agriculture
**E**nergy from **f**orage	MJ	E_{fy}	26,918	Fixed value, Bavarian State Agency for Agriculture
**E**nergy for **b**asic metabolism	MJ	E_{by}	15,300	Fixed value, Bavarian State Agency for Agriculture
**ef**ficiency factor of **con**centrates		ef_{con}	1.9	Fixed value, Bavarian State Agency for Agriculture
**p**rice for **con**centrates	€/100 kg	p_{con}	28	Fixed value, Bavarian State Agency for Agriculture
**V**ariable **C**osts for **c**alf **r**earing	€	VC_{cry}	57.66	Fixed value, Bavarian State Agency for Agriculture
**V**ariable **C**osts for **v**eterinarian	€	VC_{vy}	165	Fixed value, Bavarian State Agency for Agriculture
**V**ariable **C**osts for **wa**ter, electricity	€	VC_{way}	75.63	Fixed value, Bavarian State Agency for Agriculture
**V**ariable **C**osts for **ins**emination	€	VC_{insy}	29.41	Fixed value, Bavarian State Agency for Agriculture
**V**ariable **C**osts for **mac**hines	€	VC_{macy}	58.82	Fixed value, Bavarian State Agency for Agriculture
**V**ariable **C**osts for hired **lab**or	€	VC_{laby}	0	Fixed value, Bavarian State Agency for Agriculture
**V**ariable **C**osts **mis**celaneous	€	VC_{misy}	36.13	Fixed value, Bavarian State Agency for Agriculture

(B) Beef cattle

Variable	Unit	Notation	Calculation / value	Data source / comments
**f**attening cycle	days	f	$(w - sw)$/dwg	One fattening cycle = 22 months
**c**onversion factor to change from fattening cycle to year		c	365/f	
finishing **w**eight	kg	w	600	Fixed value, Bavarian State Agency for Agriculture
**s**tart **w**eight	kg	sw	69	Fixed value, Bavarian State Agency for Agriculture
**d**aily **w**eight **g**ain	kg/day	dwg	1.05	Fixed value, Bavarian State Agency for Agriculture
**G**ross **M**argin healthy **F**attening per **y**ear	€	GM_{Fy}	$R_{Fy} - VC_{Fy}$	
**R**evenues **F**attening per **y**ear	€	R_{Fy}	$R_{any} + R_{may}$	
**R**evenues for selling **an**imals	€	R_{any}	$pf * dw$	
slaughter **p**rice **f**attening	€/kg	pf	3.25	National yearly median of meat prices (male and female calves, cows and bulls), Federal statistical office
**d**ressed **w**eight fattening	kg	dw	$wf *$ 0.558*c	
**m**arketing costs **f**attening	€	mf	23*c	Fixed value, Bavarian State Agency for Agriculture
**f**attening **m**ortality	per cent	fm	0.031	Fixed value, Bavarian State Agency for Agriculture
**R**evenues for selling **ma**nure	€	R_{may}	53.5*c	Fixed value, Bavarian State Agency for Agriculture
**V**ariable **C**osts healthy **F**attening per **y**ear	€	VC_{Fy}	$VC_{rcy} + VC_{fey} + VC_{vy} + VC_{way} + VC_{macy} + VC_{misy}$	
**r**estocking (**c**alf purchase)	€	VC_{rcy}	145*c	Fixed value, Bavarian State Agency for Agriculture
**fe**ed (rearing, roughage, concentrates)	€	VC_{fey}	607*c	Fixed value, Bavarian State Agency for Agriculture
**v**eterinarian	€	VC_{vy}	24*c	Fixed value, Bavarian State Agency for Agriculture
**wa**ter, electricity	€	VC_{way}	19*c	Fixed value, Bavarian State Agency for Agriculture
**mac**hines	€	VC_{macy}	25*c	Fixed value, Bavarian State Agency for Agriculture
**mis**cellaneous	€	VC_{misy}	9*c	Fixed value, Bavarian State Agency for Agriculture

(C) Sheep

Variable	Unit	Notation	Calculation / value	Data source / comments
**G**ross **M**argin **h**ealthy **S**heep per **y**ear	€	GM_{Sy}	$R_{Sy} - VC_{Sy}$	
**R**evenues **S**heep per **y**ear	€	R_{Sy}	$R_{wy} + R_{any} + R_{sy}$	
**R**evenues for selling **w**ool	€	R_{wy}	$w_y * wp_y/100$	
**w**ool sold	kg	w_y	4.5	Fixed value, Mendel (2008)
**w**ool **p**rice	Cent/kg	wp_y	70	Fixed value, Mendel (2008)
**R**evenues for selling **an**imals	€	R_{any}	$R_{lamby} + R_{ewey}$	

**R**evenues for **lamb**s	€	R_{lamby}	$Rls_y + Rld_y + Rlo_y$	
**R**evenues **l**ambs **s**old (through markets)	€	Rls_y	$nl_y * pls * pl_y * wl$	
**R**evenues **l**ambs sold (**d**irectly to the consumer)	€	Rld_y	$nl_y * pld * pl_y * wl$	
**R**evenues **l**ambs (**o**wn consumption)	€	Rlo_y	$nl_y * plo * pl_y * wl$	
**n**umber of **l**ambs	n	nl_y	1.23	Fixed value, Mendel (2008)
**p**roportion of **l**ambs **s**old (traded)	per cent	pls	0.45	Fixed value, Mendel (2008)
**p**roportion of **l**ambs sold (**d**irect marketing)	per cent	pld	0.5	Fixed value, Mendel (2008)
**p**roportion of **l**ambs (**o**wn consumption)	per cent	plo	0.05	Fixed value, Mendel (2008)
**p**rice **l**amb	€/kg	pl_y	2.0	Fixed value, Mendel (2008)
average **w**eight of **l**amb	kg	wl	45	Fixed value, Mendel (2008)
**l**amb **m**ortality	per cent	cm_y	6	Fixed value, Mendel (2008)
**R**evenues for **ewe**s	€	R_{ewey}	$nes_y * pe_y * we$	
**n**umber of **s**old **e**wes	n	nes_y	$1 * (RR - eM_y)$	Fixed value, Mendel (2008)
**p**rice **e**we	€/kg	pe	0.4	Fixed value, Mendel (2008)
average **w**eight of **e**we	kg	we	90	Fixed value, Mendel (2008)
**R**eplacement **R**ate	per cent	RR	20	Fixed value, Mendel (2008)
**e**we **M**ortality	per cent	eM_y	2	Fixed value, Mendel (2008)
**R**evenues for selling **s**kin	€	R_{sy}	$ns_y * ps_y/100$	
**n**umber of **s**kins sold (direct marketing)	n	ns_y	$nl_y * pld$	
**p**rice **s**kin	€/skin	ps_y	3	Fixed value, Mendel (2008)
**V**ariable **C**osts healthy **S**heep per **y**ear	€	VC_{Sy}	$VC_{ry} + VC_{fey} + VC_{vy} + VC_{way} + VC_{insy} + VC_{mary} + VC_{laby} + VC_{misy}$	
**V**ariable **C**osts for **r**estocking	€	VC_{ry}	0	Fixed value, Mendel (2008)
**V**ariable **C**osts for **fe**ed	€	VC_{fey}	96	Fixed value, Mendel (2008)
**V**ariable **C**osts for **v**eterinarian	€	VC_{vy}	5.50	Fixed value, Mendel (2008)
**V**ariable **C**osts for **wa**ter, electricity, bedding	€	VC_{way}	5	Fixed value, Mendel (2008)
**V**ariable **C**osts for **ins**emination (ram)	€	VC_{insy}	4	Fixed value, Mendel (2008)
**V**ariable **C**osts for **mar**keting	€	VC_{mary}	12.80	Fixed value, Mendel (2008)
**V**ariable **C**osts for hired **lab**or	€	VC_{laby}	7	Fixed value, Mendel (2008)
**V**ariable **C**osts **mis**cellaneous	€	VC_{misy}	4.5	Fixed value, Mendel (2008) e.g. animal health insurance, herding dog.

Table S2: Variables used in Gross Margin Analysis influenced by BTV-8 in dairy cattle (A), beef cattle (B), and sheep (C) per animal per year, if clinical signs occurred. Normal distributions denote a mean value μ and the standard deviation σ in brackets; Triangle distributions denote a distribution with lower and upper limits and mode. Bold letters in the variables are used in the notations. To avoid duplicate notations, we combined upper and lower case characters.

(A) Dairy cattle

Variable	Unit	Notation	Impact of BTV-8	Data source / comments
Reduced **m**ilk **y**ield	kg/cow	my_{BTy}	Normal distribution (μ=100; σ=100)	Based on Heimberg et al. (2008)
Increased **C**alving **i**nterval (additional number of days)	days	Ci_{BT}	Normal distribution (μ=80; σ=100)	Based on Heimberg et al. (2008)
Increased **c**alf **m**ortality rate	per cent	cm_y	3	Fixed value, based on Heimberg et al. (2008)
Increased **M**ortality rate adult **C**ow	per cent	CM_y	Triangle distribution (0; 1; 2)	The value of animals that succumbed to disease as well as costs for culling and rendering were not included in the GMA, but in the model run on the national level.
Increased **R**eplacement **R**ate	per cent	RR	5	Fixed value, based on Heimberg et al. (2008)
Increased **V**ariable **C**osts for **v**eterinary treatment **d**airy	€	VC_{vdy}	Triangle distribution (0; 5; 40)	Based on Heimberg et al. (2008)

(B) Beef cattle

Variable	Unit	Notation	Impact of BTV-8	Data source / comments
Reduced **d**aily **w**eight **g**ain	kg/day	dwg	Normal distribution (μ=-0,1; σ=0, 01);	Fixed value, expert opinion
Increased **V**ariable **C**osts for **v**eterinary treatment **f**attening	€	VC_{vfy}	Normal distribution (μ=10; σ=1)	Fixed value, expert opinion

(C) Sheep

Variable	Unit	Notation	Impact of BTV-8	Data source / comments
**R**evenues for selling **an**imals	€	R_{any}	$R_{lamby} + R_{ewey}$	
**n**umber of **l**ambs	n	nl_y	0.7	Fixed value, expert opinion
Increased **l**amb **m**ortality	per cent	lm_y	Normal distribution (μ=25; σ=4)	Expert opinion, results of outbreak investigations
Reduced **w**eight of **e**we	kg	we	15	Fixed value, expert opinion, results of outbreak investigations
Increased **e**we **m**ortality	per cent	em_y	Normal distribution (μ=20; σ=4)	Expert opinion, results of outbreak investigations
Increased **R**eplacement **R**ate	per cent	RR	10	Fixed value, expert opinion, result of outbreak investigations
Increased **V**ariable **C**osts for **v**eterinary treatment **s**heep	€	VC_{vsy}	Normal distribution (μ=10; σ=4)	Fixed value, expert opinion

Gethmann, J., Probst, C., Fröhlich, A., Ziller, M., Staubach, C., Conraths, F.J., 2011. Evaluation of Bluetongue surveillance in Germany. Epidémiol. et santé anim. 59-60, 159.

Heimberg, P., Adam, F., Holsteg, M. 2008. Blauzungenerkrankung bei Rind, Schaf und Ziege – Klinik, Schäden Verluste (Landwirtschaftskammer NRW, Tiergesundheitsdienst).

Kloepfer, F., Pikart-Müller, M., Sauer, N., Schroers, J.O., 2012. Betriebsplanung Landwirtschaft 2012/2013, Vol 23. Kuratorium für Technik und Bauwesen in der Landwirtschaft e.V. (KTBL).

Mendel, C., 2008. Praktische Schafhaltung. Ulmer Eugen Verlag, 264 p.

Table S3: Variables used in the economic model to estimate the financial impact of BTV-8 per year at the national level. Triangle distributions denote a distribution with lower and upper limits and mode. Bold letters in the variables are used in the notations. To avoid duplicate notations, we combined upper and lower case characters.

Variable	Unit	Notation	Calculation	Data source / comments
Total **C**osts incurred by **BT**V-8	€	C_{BT}	$\sum_{y=2006}^{2018} DC_y + IC_y$	
**D**irect **C**osts per **y**ear	€	DC_y	$DCc_y + DCd_y$	
**D**irect **C**osts due to **c**linical illness per **y**ear	€	DCc_y	$DCDc_y + DCFc_y + DCSc_y$	
**D**irect **C**osts **D**airy due to **c**linical illness	€	$DCDc_y$	$nCi_y * rcc * pd/100 * dC_{py}$	
**D**irect **C**osts **F**attening due to **c**linical illness	€	$DCFc_y$	$nCi_y * rcc * pf/100 * dC_{py}$	
**D**irect **C**osts **S**heep due to **c**linical illness	€	$DCSc_y$	$nSi_y * rsc * dC_{py}$	
Total **n**umber of **a**nimals (**c**attle)	N	nac	12.5 - 12.9 million	Fixed annual values; Federal statistical office
Total **n**umber of **a**nimals (**d**airy)	n	nad	4.1 - 4.3 million	Fixed annual values; Federal statistical office
Total **n**umber of **a**nimals (**f**attening)	n	naf	1.5 - 2.1 million	Fixed annual values; Federal statistical office
Total **n**umber of **f**arms (**c**attle)	n	nfc	139 - 188 thousand	Fixed annual values; Federal statistical office
Total **n**umber of **f**arms (**d**airy)	n	nfd	63 - 106 thousand	Fixed annual values; Federal statistical office
Total **n**umber of **a**nimals (**s**heep)	n	nas	2.4 million	Fixed value; Federal statistical office
Total **n**umber of **f**arms (**s**heep)	n	nfs	29 thousand	Fixed value; Federal statistical office
**p**roportion of **d**airy cows in the cattle population	per cent	pd	$\frac{nad}{nac}$	
**p**roportion of **f**attening animals in the cattle population	per cent	pf	$\frac{naf}{nac}$	
**d**irect **C**osts **D**airy per animal	€	dCD_y	$GM_{Dy} - GM_{cpy}$	Distribution of direct costs previously calculated in the GMA (see Tables S1-S2).
**d**irect **C**osts **F**attening per animal	€	dCF_y	$GM_{Fy} - GM_{cpy}$	
**d**irect **C**osts **S**heep per animal	€	dCS_y	$GM_{Sy} - GM_{cpy}$	
**G**ross **M**argin of a **c**linically ill animal (per species/**p**roduction type)	€	GM_{cpy}	See supplementary tables S1 and S2	
**n**umber of **C**attle newly **i**nfected	n	nCi_y	$\frac{I_{sy}}{100} * ncz_y$	
**n**umber of **S**heep newly **i**nfected	n	nSi_y	$\frac{I_{sy}}{100} * nsz_y$	
**I**ncidence per **s**pecies (cattle, sheep)		I_{sy}	$\frac{I_{2006}}{P_{2006}} * P_{sy}$	

Farm **P**revalence: number of officially reported outbreaks per **s**pecies	n	P_{sy}		German animal disease notification system, *TierSeuchenNachrichten*; https://tsn.fli.de/; public site: https://tsis.fli.de
**n**umber of **c**attle in restriction **z**ones	n	ncz_y	fixed values	2006: cattle/ sheep population in the affected federal states, according to the Federal statistical office and the cross-sectional study performed in 2007 (Gethmann et al., 2011)<br>2007-2011: whole German cattle/ sheep population according to the Federal statistical office;<br>2012-2018: zero animals (no restriction zones)
**n**umber of **s**heep in restriction **z**ones	n	nsz_y	fixed values	
Morbidity **r**ate **c**attle (proportion of infected cattle that show **c**linical signs)	per cent	rcc	Triangle distribution (0.05; 0.1; 0.15)	Based on data obtained from the German animal disease notification system
Morbidity **r**ate **s**heep (proportion of infected sheep that show **c**linical signs)	per cent	rsc	Triangle distribution (0.15; 0.2; 0.25)	Based on data obtained from the German animal disease notification system
**D**irect **C**osts through **d**eath per **y**ear	€	DCd_y	$DCCd_y + DCSd_y$	
**D**irect **C**osts **C**attle through **d**eath	€	$DCCd_y$	$nCd_y * vc_y$	
**D**irect **C**osts **S**heep through **d**eath	€	$DCSd_y$	$nSd_y * vs_y$	
**n**umber of **d**ead **C**attle	n	nCd_y	$nCI_y * nCI_{2007}/nCd_{2007}$	Estimation based on the Animal Disease Compensation Funds of the federal states for 2007. It is assumed that the mortality at the population level remained constant throughout the years.
**n**umber of **d**ead **S**heep	n	nSd_y	$nSI_y * nSI_{2007}/nSd_{2007}$	
**v**alue of dead **c**attle	€/animal	vc_y	Triangle distribution (1,500; 1,700; 1,900)	Mean compensation paid to the farmers by the Animal Disease Compensation Fund of the federal state of North-Rhine Westphalia (cattle: includes animal value and 200 € disposal costs; sheep: includes animal value)
**v**alue of dead **s**heep	€/animal	vs_y	Triangle distribution (120; 145; 170)	
**I**ndirect **C**osts per **y**ear	€	IC_y	$ICS_y + ICE_y + ICI_y + ICV_y + ICM_y + ICA_y$	
**I**ndirect **C**osts for BT **S**urveillance	€	ICS_y	$nfs_y * (ct_f + cp_f) + ns_{sy} * csa_s + n_{ELI} * c_{ELI} + n_{PCR} * c_{PCR}$	
**n**umber of tested **f**arms for **s**urveillance	n	nfs_y		Reports of the federal states to the Friedrich-Loeffler-Institut
**c**osts for **t**ravelling per tested **f**arm	€/farm	ct_f	$k * d * 2$	Sampling for both the sentinel and the cross-sectional study (Gethmann et al., 2011) was done by official veterinarians and not invoiced according to the veterinary fee schedule (*Gebührenordnung für Tierärzte; https://www.bundestieraerztekammer.de/tieraerzte/beruf/got/*). Therefore, for these two programs, travel costs and personnel costs were estimated and added separately. Travel costs were multiplied by 2 to account for the full round-trip time.
Fee charged per **k**m	€/km	k	Triangle distribution (0.30; 0.33; 0.36)	
Average **d**istance veterinary office - farm	km	d	Triangle distribution (5; 10; 20)	
**c**osts for **p**ersonnel per tested **f**arm	€/farm	cp_f	$ts_f * cp_h$	
**t**ime **s**pent at **f**arm	h/farm	ts_f	Triangle distribution (0.5; 1; 1.5)	
**c**osts for **p**ersonnel per **h**our	€/hour	cp_h	Triangle distribution (70; 77; 84)	
**n**umber of **s**amples per **s**pecies and **y**ear	n	ns_{sy}		

**c**osts for **sa**mpling per **s**pecies (cattle, sheep)	€/animal	csa_s	Triangle distribution (1.5; 2; 2.5)	Monthly reports of the federal states to the German Federal Ministry for Food and Agriculture and annual applications of the Federal Ministry to the European Commission for co-financing (https://ec.europa.eu/food/funding/animal-health/national-veterinary-programmes_en)
**n**umber of **s**amples per **s**pecies tested with **ELI**SA and **PCR**		ns_{sELI} ns_{sPCR}		
**c**osts for **ELI**SA and **PCR**	€/test	c_{ELI} c_{PCR}	ELISA: 1.69 – 4.94<br>PCR: 17.12 - 25.08	Annual applications of the Federal Ministry for Food and Agriculture to the European Commission for co-financing. Includes tests in regional and national reference laboratories, i.e. laboratory analysis, test kits and chemicals (https://ec.europa.eu/food/funding/animal-health/national-veterinary-programmes_en)
**I**ndirect **C**osts for additional measures for **E**xport	€	ICE_y	$cce_y + cse_y$	
**c**osts for measures **c**attle **e**xport	€	cce_y	$nce_y * pe_y * cet_y$	
**n**umber of **c**attle **e**xported	n	nce_y	fixed values	Eurostat (as of 08.01.2018), excluding BT-affected countries (Belgium, Switzerland, Luxembourg, The Netherlands, France) (http://epp.eurostat.ec.europa.eu/newxtweb/)
**p**roportion of animals **e**xported to BT-free countries (additional measures necessary)	per cent	pe_y	pe_{2006} =Triangle distribution (15; 20; 25)<br>pe_{2007} =Triangle distribution (60; 70; 80)<br>pe_{2008} =Triangle distribution (80; 90; 100)<br>pe_{2009} =Triangle distribution (30; 40; 50)	Annual applications of the Federal Ministry for Food and Agriculture to the European Commission for co-financing. In 2006, the epidemic did not start before August. The annual mean proportion was therefore rather low; from 2010 onwards all animals were vaccinated, so testing was no longer necessary.
**c**osts for **e**xport **t**est	€/animal	cet_y	Triangle distribution (20; 25; 50)	Test costs (c_{PCR}) as reported by the Federal States
**c**osts for measures **s**heep **e**xport	€	cse_y	$nse_y * pe_y * cet_y$	
**n**umber of **s**heep **e**xported	n	nse_y	fixed values	Eurostat (as of 08.01.2018), excluding BT-affected countries (Belgium, Switzerland, Luxembourg, The Netherlands, France) (http://epp.eurostat.ec.europa.eu/newxtweb/)
**I**ndirect **C**osts for treatment with **I**nsecticides	€	ICI_y	$cci_y + csi_y$	
**c**osts for **c**attle **i**nsecticide treatment	€	cci_y	$(ncz_y * ci_y + ncfz_y * ci_{fy}) * pic_y$	
**n**umber of **c**attle in restriction **z**ones	n	ncz_y	fixed values	For 2006: Results of a cross-sectional study (Gethmann et al., 2011); for 2007-2011: all animals
**c**osts for **i**nsecticides	€/animal	ci_y	Triangle distribution (1; 1.3; 1.6)	Market prices (e.g. Butox® pour on: Price for 2500 ml ~ 124 €, ~80-120 cattle can be treated → 1.0-1.6 €/cattle)

**n**umber of **c**attle **f**arms in restriction **z**ones	n	$ncfz_y$	fixed values	For 2006: cattle farms of the affected federal states (Gethmann et al., 2011); For 2007-2011: all cattle farms in Germany (Federal statistical office); 2012-2018: zero farms (no restriction zones).
**c**osts for **i**nsecticide treatment per **f**arm (personnel)	€/farm	ci_{fy}	Triangle distribution (10; 20; 30)	Mean farm size ~ 60 animals, time per animal ~ 0.5-1 minute
**p**roportion of **i**nsecticide treated **c**attle	per cent	pic_y	pic_{2006} = 0.8; pic_{2007} = 0.4; pic_{2008} = 0.1 $pic_{2009-2012}$ = 0.05	Own estimate; in 2006, most farmers used insecticides; in the following years, insecticide treatment decreased.
**c**osts for **s**heep **i**nsecticide treatment	€	csi_y	$(nisf_y * s_f * ci_y) + (nisf_y * cif_y)$	
**n**umber of **i**nfected **s**heep **f**arms	n	$nisf_y$		German animal disease notification system
Mean number of **s**heep per **f**arm	n	s_f	$\frac{nas}{nfs}$	
**I**ndirect **C**osts for **V**accination	€	ICV_y	$ICVc_y + ICVs_y$	
**I**ndirect **C**osts for **V**accination of **c**attle	€	$ICVc_y$	$nvdc_y * (cvc_d + cvac_d) + (nvcf_y * cc_f)$	
**n**umber of **v**accine **d**oses **c**attle	n	$nvdc_y$	fixed	Identification and Information System for Animals
**n**umber of **v**accinated **c**attle **f**arms	n	$nvcf_y$	fixed	Identification and Information System for Animals
**c**osts for **v**accine **c**attle	€/dose	cvc_d	0.62	Veterinary fee schedule; data source for 2009: Annual application of the BMEL to the European Commission for co-financing
**c**osts for **vac**cination **c**attle	€/dose	$cvac_d$	Triangle distribution (1; 1.4; 1.8)	Animal Disease Compensation Fund of the federal states; veterinary fee schedule. Fees charged by veterinarians for vaccinations were 3.44 €/cattle for the first five animals and 2.30 €/cattle for all other animals. However, the annual applications for co-financing reported about 1.45 € per vaccination dose. This was possibly due to special conditions for BT mass-vaccination in some federal states and because vaccination was in some districts done by official veterinarians who did not charge extra.
**c**osts per vaccinated **c**attle **f**arm	€/farm	cc_f	Triangle distribution (17; 19; 23)	Veterinary fee schedule. Herd fee charged by veterinarians per callout to a cattle farm including travel costs and veterinary advice
**I**ndirect **C**osts for **V**accination of **s**heep	€	$ICVs_y$	$nvs_y * (cvs_d + cvas_d) + (nvsf_y * cs_f)$	
**n**umber of **v**accinated **s**heep	n	nvs_y	fixed	Identification and Information System for Animals. In contrast to cattle, sheep were vaccinated only once per year
**n**umber of **v**accinated **s**heep **f**arms	n	$nvsf_y$	fixed	Identification and Information System for Animals
**c**osts for **v**accine **s**heep	€/dose	cvs_d	0.447	
**c**osts for **va**ccination **s**heep	€/dose	$cvas_d$	Triangle distribution (1.35; 1.50; 1.65)	Animal Disease Compensation Fund of the federal states; veterinary fee schedule: Fees charged by veterinarians for vaccinations were 1.14 €/sheep. However, the annual applications for co-financing reported about 1.45 € per

				vaccination dose. This was possibly due to special conditions for BT mass-vaccination in some federal states and because vaccination was in some districts done by official veterinarians who did not charge extra.
**c**osts per vaccinated **s**heep **f**arm	€/farm	cs_f	Triangle distribution (15; 17; 19)	Veterinary fee schedule. Herd fee charged by veterinarians per callout to a sheep farm including travel costs and veterinary advice.
**I**ndirect **C**osts for vector **M**onitoring	€	ICM_y	$nvt_y * (cvmt_y + cvmm_y) + nvme_y * cvme_y$	
**n**umber of **v**ector **t**raps	n	nvt_y	fixed	Application tables for co-financing 2007 and 2008
**c**osts **v**ector **m**onitoring (**t**raps)	€/trap	$cvmt_y$	821 (2007), 770 (2008)	Costs for traps and data loggers, applications of the Federal Ministry for Food and Agriculture to the European Commission for co-financing
**c**osts **v**ector **m**onitoring (trap **m**anagement)	€/trap	$cvmm_y$	1000	Fixed value, expert opinion of Dr. Helge Kampen, Friedrich-Loeffler-Institut
**n**umber of **v**ector **m**onitoring (**e**ntomological tests)	n	$nvme_y$	fixed	Application tables for co-financing 2007 and 2008
**c**osts **v**ector **m**onitoring (**e**ntomological tests)	€/sample	$cvme_y$	fixed	Application tables for co-financing 2007 and 2008
**I**ndirect **C**osts for reporting and **A**dministration	€	ICA_y	$tP_y * d * k * 2 + (t_f * p_h)$	Collation, standardization and reporting data
**t**otal BT **P**revalence (number of affected cattle and sheep farms)	n	tP_y	fixed values	German animal disease notification system
Average **d**istance veterinary office - farm	km	d	Triangle distribution (5; 10; 20)	Estimated based on the size of the districts (Federal statistical office)
Fee charged per **k**m	€/km	k	Triangle distribution (0.30; 0.33; 0.36)	Based on official mileage allowance (*„Einkommensteuergesetz (EStG) § 9 Werbungskosten“*)
Average **t**ime spent per **f**arm	hours/ farm	t_f	Triangle distribution (1.2; 2.2; 2.75)	Estimated based on own experience
Average **p**ersonnel costs per **h**our	€/hour	p_h	Triangle distribution (70; 77; 84)	Based on labour cost rates of the Federal Ministry of Finance (https://www.bundesfinanzministerium.de/Content/DE/Standardartikel/Themen/Oeffentliche_Finanzen/Bundeshaushalt/personalkostensaetze.html)
